# Eugenol-Rich Essential Oil from *Pimenta dioica*: In Vitro and In Vivo Potentialities against *Leishmania amazonensis*

**DOI:** 10.3390/ph17010064

**Published:** 2023-12-29

**Authors:** Lianet Monzote, Laura Machín, Adiel González, Ramón Scull, Yamilet I. Gutiérrez, Prabodh Satyal, Lars Gille, William N. Setzer

**Affiliations:** 1 Parasitology Department, Center of Research, Diagnostic and Reference, Institute of Tropical Medicine “Pedro Kouri”, Havana 17100, Cuba; 2 Research Network Natural Products against Neglected Diseases (ResNetNPND), 48149 Munster, Germany; 3 Department of Pharmacy, Institute of Pharmacy and Food, Havana University, Havana 13600, Cuba; 4 Aromatic Plant Research Center, 230 N 1200 E, Suite 100, Lehi, UT 84043, USA; psatyal@aromaticplant.org; 5 Institute of Pharmacology and Toxicology, Department of Biomedical Sciences, University of Veterinary Medicine, Veterinärplatz 1, A-1210 Vienna, Austria; lars.gille@vetmeduni.ac.at; 6 Department of Chemistry, University of Alabama in Huntsville, Huntsville, AL 35899, USA

**Keywords:** *Pimenta dioica*, essential oil, eugenol, leishmania, BALB/c, cutaneous leishmaniasis

## Abstract

*Pimenta dioica* L. is one the most recognized species with diverse biological activities. In this study, in vitro activity and in vivo efficacy of essential oil from *P. dioica* (EO-Pd) was evaluated. The main compound was also included in the animal studies and its in silico prediction related to biological activities, molecular ligands, drug likeness, and ADME (absorption, distribution, metabolism, and excretion) properties are listed. The chemical composition analyzed by GC-MS retrieved 45 components, which the most abundant compound was the eugenol (80.1%). The EO-Pd was able to inhibit the growth of *L. amazonensis* (IC_50_ = 9.7 ± 0.7 and 11.3 ± 2.1 µg/mL, promastigotes and amastigotes, respectively). The cytotoxicity assay showed a CC_50_ of 104.5 ± 0.9 µg/mL and a selectivity index of 9. In the model of cutaneous leishmaniasis in BALB/c mice, the effect of EO-Pd and eugenol was observed after treatment at 30 mg/kg by intralesional route with 5 administrations every 4 days. In the in silico predictions, some targets that justified the antileishmanial activity of eugenol and good drug like properties for this compound, were obtained. This study showed for first time the potential of EO-Pd to inhibit *L. amazonensis,* which could be linked to the activity of major compound eugenol.

## 1. Introduction

*Pimenta dioica* L. (Family Myrtaceae) is a plant characterized by its high essential oil (EO) content, which has been used in worldwide traditional medicine to alleviate the symptoms of microbial infections, including Caribbean region [1,2]. During the last decade, some biological activities have been demonstrated for essential oil of *P. dioica* (EO-Pd), including antioxidant [3], antimicrobial [4] and antiparasitic, which have been described against ectoparasites [5], mosquitoes [6] and pinewood nematode [7]. Among the most important component isolated from the EO-Pd, eugenol has been described, which composes 60–90% of the oil [8]. In general, the described biological effects of EO-Pd have been explained on assumption of the bioactive compound eugenol [8].

There have been no previous reports regarding the activity of EO-Pd against the *Leishmania* parasite. However, some reports have shown the potentialities of EOs rich in eugenol (EO-RE) against different *Leishmania* species [9,10]. Currently, leishmaniasis is a neglected tropical disease endemic primarily to low- and middle-income countries, for which there has been inadequate development of affordable, safe, and efficacious therapies [11]. Cutaneous leishmaniasis (CL) is the most common presentation of leishmaniasis, with global estimates of 0.7 to 1.2 million cases per year. Available antileishmanial therapies to CL are significantly limited (low efficacy, toxicity, adverse side effects, drug resistance, length of treatment, and cost), so there is an urgent need to discover new compounds. Therefore, various successful approaches have been explored to search for antileishmanial drugs as therapeutic compounds and natural products may also contribute to the development of new drugs based on their chemical structures [12,13]. In recent years, an increased interest in EOs as alternative therapies in the treatment of leishmaniasis has been presented in original articles and reviewed as promising natural products [14,15].

Following our program of investigation related with new antileishmanial agents, we evaluated the potential in vitro activity and in vivo efficacy of EO-Pd. In addition, the main compound was also evaluated against the experimental CL, as well as, its in silico predictions with respect to biological activities, molecular ligands, drug likeness and ADME properties, are also listed.

## 2. Results

### 2.1. Chemical Characterization of EO

To characterize the EO-Pa, the chemical composition was analyzed by GC-MS. Detected compounds are listed in Table 1, in which 45 components were identified representing 99.7% of EO. The chromatogram is shown in Supplementary Figure S1. In this oil, the phenolic compounds were the most abundant with 85.1%, follow by 6.5% of monoterpenes hydrocarbons, 3.6% of oxygenated monoterpenoids, 2.6% of oxygenated sesquiterpenoids, 1.8% of sesquiterpene hydrocarbons and 0.1% of other components type. In correlation with previous reports, the most abundant compound was eugenol (80.1%).

**Figure 1:**
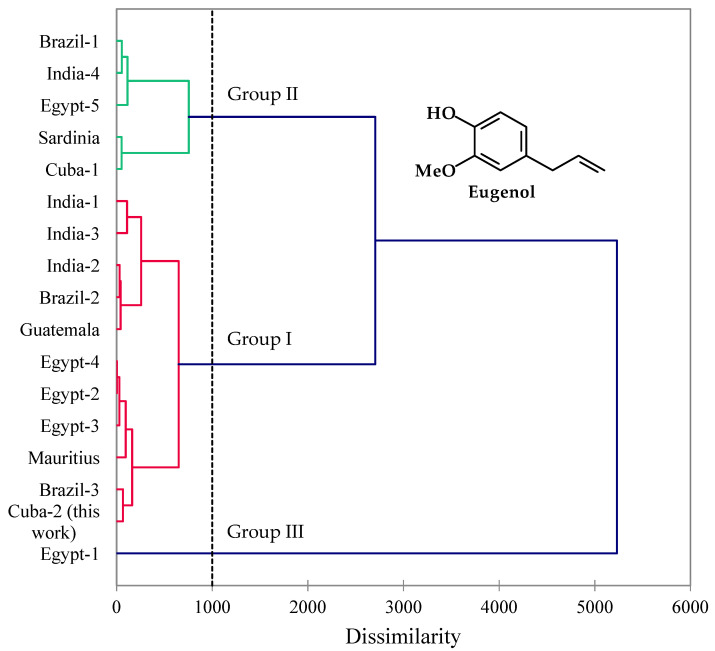
Dendrogram obtained from the hierarchical cluster analysis of *Pimenta dioica* leaf essential oil compositions.

Agglomerative hierarchical cluster analysis (HCA) is a common type of clustering used to group objects in clusters based on their similarities. The method starts by treating individual data points as a single cluster, then it is continuously merged based on similarity until one large cluster containing all the objects is obtained. The result is a hierarchical tree (dendrogram) reflecting the dissimilarities of the objects. In this study, HCA was carried out using the concentrations of the most abundant chemical components of EO-Pd from this work and 16 previously reported compositions (Figure 1). The HCA shows three well-defined groupings based on chemical compositions. Group I is dominated by eugenol (65.8–88.8%), Group II has lower concentrations of eugenol (44.5–55.5%) as well as relatively high concentrations of myrcene (0.1–22.4%), and Group III, a single sample, is low in eugenol (8.6%), but rich in myrcene (44.1%), 1,8-cineole (18.8%), and limonene (11.7%). Thus, the EO-Pd in this work falls into the eugenol-rich Group I cluster.

### 2.2. In Vitro Assay

The effect of EO-Pd on growth of promastigotes and intracellular amastigotes of *L. amazonensis* was quantitatively determined. On both parasite forms, EO-Pd caused a similar IC_50_ (*p* > 0.05); which was statistically (*p* < 0.05) superior to Pentamidine^®^ (Table 2). However, SI retrieved the same value, since CC_50_ effect of EO-Pd was 10 times higher (*p* < 0.05) in comparison with Pentamidine^®^.

### 2.3. In Vivo Assay

**Figure 2:**
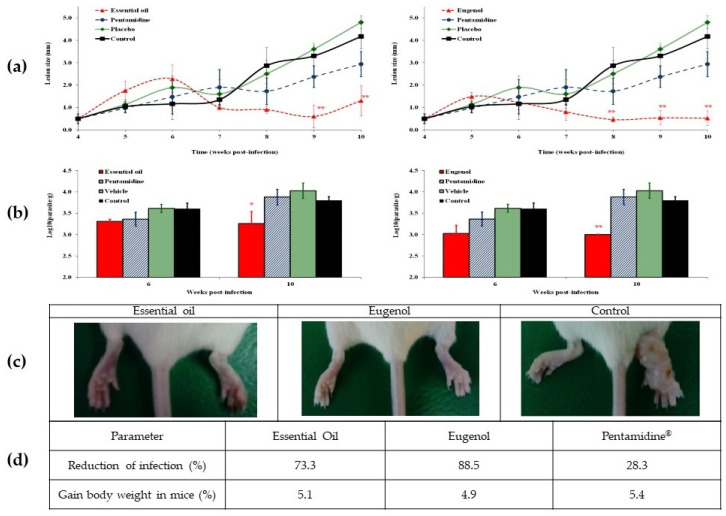
Effect of essential oil from *Pimenta dioica* in BALB/c mice infected with *Leishmania amazonensis*. (**a**) The results of lesion size and (**b**) parasite burden are expressed as mean ± standard deviation. (**c**) Photographs of footpads at week 10 post-infection. At this time, (**d**) percent of reduction of infection was calculated (multiply lesion size and parasite burden of treated group divided by same data of untreated animals). Statistical differences with respect to placebo, untreated groups, and the reference drug Pentamidine^®^ are represented as * (*p* < 0.05) and ** (*p* < 0.01).

In addition to in vitro experiments, an in vivo evaluation of the EO-Pd and eugenol after administration by intralesional route was also carried out. In the groups treated with these products, infection control was observed (Figure 2a,b). However, eugenol displayed a quick and efficient decrease of lesion size and parasite burden (*p* < 0.01) compared with animals treated with placebo or Pentamidine^®^, as well as with respect to untreated mice. The higher efficacy of eugenol can be appreciated in photographs of infected and uninfected footpads (Figure 2c) and after reduction of infection calculation (Figure 2d). In addition, throughout the observation period (10 weeks p.i.), animals showed no death or signs of toxicity and increase in weight body (Figure 2d).

### 2.4. In Silico Predictions

The main compound of EO-Pd, eugenol, was used for PASS and ADME prediction studies using web tools. Table 3 presents the results of PASS prediction study, in which seven different activities and 31 ligand interactions were obtained with *Pa* > 0.7. The analysis of bioavailability and ADME properties of eugenol are compiled in Table 4. Physicochemical parameters used to predict drug-likeness properties showed that comply the rules (Lipinski or rule of five (RO5), Ghose, Veber and Egan); except to Muegge rule with one violation. In general, drug likeness values of eugenol compounds were same or better than pentamidine.

## 3. Discussion

*Pimenta* is an important myrtaceous genus that encompasses 15 species, mostly found in the American Caribbean area, and commonly used for several medicinal purposes [16,17]. *Pimenta dioica* is one the most recognized species from this genus, which has been used to relieve indigestion in Cuban traditional medicine [18]. However, the widespread and diverse uses of this plant, have stimulated several studies in the last two decades on systematic investigations of the potential evidence-based medical use by the population that have been inherited from ancestors among different cultures. With this finding, more scientific studies are necessary to delineate the potential medicinal value of *P. dioica*, their main constituents or enriched products [8].

This research article presents the chemical composition analysis of the leaf essential oil of *P. dioica* (EO-Pd) grown in NBG located in Havana, Cuba. The chemical profile of the studied oil was generally consistent with those previously reported in literature from Jamaica [7], India [3,6], Mauritius [19] and Egypt [2], which eugenol was highlighted as the main component. Nevertheless, a fruit essential oil from the Dominican Republic [20] showed a higher percent of methyleugenol. In Mexico, both methyleugenol and eugenol chemotypes of the fruit essential oil have been reported [4,5]. As shown in Figure 2, the eugenol-rich EO-Pd constitutes the most common chemotype for the leaf essential oil, although it is likely that chemical composition of EO-Pd depends on geographical location, climatic and edaphic conditions, seasonality, as observed in other EOs [21,22].

The study of in vitro antileishmanial potentialities of the EO-Pd showed good results related with it activity and selectivity. In the scientific literature, EO-RE from other plants has been also shown potential in vitro antileishmanial activity. For example, EO-RE from *Syzygium aromaticum* L. (eugenol content = 59.7%) cause an IC_50_ of 21 µg/mL and 15.24 µg/mL against promastigotes and intracellular amastigotes of *L. donovani*, respectively, and presented no adverse cytotoxic effects against murine macrophages at 200 µg/mL [9]. Le and collaborators reported that EO-RE from *Ocimum gratissimum* L. (eugenol content = 86.5%) displayed an IC_50_ of 4.85 nL/mL against *L. mexicana* without cytotoxicity at the highest tested concentration (50 nL/mL) on WI38 and J774 mammalian cells [10]. Nevertheless, only a few published studies have demonstrated that EO-RE have antiparasitic activity [23], which constitutes a contribution of this present study.

On the other hand, the profile of in vitro antileishmanial activity of eugenol was summarized by Le and collaborators in 2017. In the cited study, the pure compound was effective on promastigotes of *L. amazonensis* and *L. mexicana* with an IC_50_ value of 12.7 µg/mL [24] and 2.72 µg/mL [25], respectively. In this study, values in same range were obtained for EO-Pd (IC_50_ = 9.7 µg/mL). However, contradictory results (did not display activity) were described for different *L. infantum*/*chagasi* strains [24], causal agents of Visceral Leishmaniasis (VL). It is known that for leishmaniasis, specificities related to individual species should be considered, in particular with respect to variation in drug susceptibility between CL and VL species [26].

Although international criteria to develop natural products against *Leishmania* are not clear at all, specifically the proposed route map for the discovery and pre-clinical development of new drugs for CL treatment recommend that products should be able to kill amastigotes of parasite (E_max_ = 90%) and show a SI > 5 [26]. Then, biological in vitro parameters obtained for EO-Pd justify the subsequent exploration on animal models. This led us to investigate the effect of EO-Pd and eugenol on experimental CL.

The majority of CL cases in humans are uncomplicated, with localized single lesions. Available drugs are often administered via systemic routes, exposing patients to unacceptable levels of systemic toxicity. Currently, for New World CL, most patients are treated by systemic route, even though it has already been recommended the use of intralesional treatment by Pan-American Health Organization [27], with the aim to reduce side effects and a rapid circulation clearance [27,28]. In addition, an accelerated healing of the cutaneous lesions and a high percentage of cure with lower cost are also benefits compared to conventional therapy [28].

The efficiency of EO-Pd and eugenol was observed after treatment of BALB/c mice infected with *L. amazonensis* by intralesional route at 30 mg/kg. Both groups treated with the studied products, showed remarkably smaller CL lesions in comparison with control animals: (i) with no treatment; (ii) treated with placebo; and (iii) treated with reference drug. In addition, the *Leishmania* parasite burden, measured by microtitration method, also displayed significant differences between tested products with control groups. Finally, reduction of infection (taking into account the lesion size and the parasite burden compared to untreated animals) demonstrated the superior activity by both tested products compared with reference drugs.

It is important to mention that, in this study, the treatment was initiated when the infection has already been established (week four post-infection), which the disease in untreated animal has already progressed to a stage where ulcers and scars can be avoided. In contrast, treated mice with EO-Pd and eugenol displayed a complete epithelization of the skin (as shown in Figure 2c). Secondly, a single dose of 30 mg/kg the EO-Pd or eugenol was administered, although this is the first time that the effect of these products is evaluated. In this sense, Caridha and collaborators suggest using a maximum dose of 30 mg/kg as part of the route map for the discovery and pre-clinical development of new drugs for CL cutaneous leishmaniasis [26], and we have been obtained promising results of Cuban EOs administered to experimentally infected mice with *L. amazonensis*, such as the EO from *Artemisia absinthium* L. [29], *Melaleuca leucadendra* L. [30], and *Pluchea carolinensis* (Jacq.) G. Don. [31]. These results could suggest that in vitro results were potentially correlated with the in vivo studies in BALB/c mice infected with *L. amazonensis*, a species that causes difficult-to-treat CL in Latin America [32].

Other generic relevant international criteria for drug development against leishmaniasis, suggest that a potential product should display no acute toxicity in the animal studies [33]. In this study, although no formal in vivo safety studies were performed at this stage, careful observation could be informative. In this sense, the animals were also checked daily, for signs of morbidity and death according to the international guidelines (WHO/OECD), which increase of body weight were observed and manifestation of toxicity was not observed. Nevertheless, toxicity studies should be designed related to safety of evaluated products, such as acute toxicity of EO-Pd and eugenol.

In particular, in the animal model, eugenol showed higher efficacy than the EO-Pd. For centuries, natural substances from plants have been used to control and treat diseases. However, many large pharmaceutical companies have been focused on pure compounds isolated from plants to develop modern pharmaceutical agents or generate large libraries of synthetic compounds to the detriment of natural products-based drug discovery research [34,35]. Eugenol is a phenylpropanoid largely used as flavoring agent for food and displays many pharmacological actions [23]. In particular, the observed antileishmanial action of eugenol could be attributed to several mechanisms. Furthermore, pharmacological properties and probable cellular ligands were analyzed using in silico prediction programs using web tools.

In the obtained activities by PASS analyses, antiprotozoal/antiparasitic effects were not listed, although some of them could contribute to skin diseases healing, including CL. Among then, antieczematic (*Pa* = 0.868), mucomembranous protector (*Pa* = 0.835) and antiseptic (*Pa* = 0.722) could be mentioned. However, when ligand interaction prediction was analyzed, several targets for the antileishmanial activity were obtained. First one, related with mitochondrial inhibition (ubiquinol-cytochrome-c reductase inhibitor: *Pa* = 0.825), which experimental data demonstrated that eugenol-rich EO from *S. aromaticum* cause mitochondrial membrane potential and reactive oxygen species generation [9]. Other relevant ligands showed relation with membrane fluidity (membrane permeability inhibitor: *Pa* = 0.781) and apoptosis promotion (apoptosis agonist: *Pa* = 0.743 and MAP kinase stimulant: *Pa* = 0.735), mechanisms recently proposed by Hughes et al., (2023) in promastigotes of *L. mexicana* [25]. The exact mechanism of the antiparasitic action of eugenol is unknown; although has been studied on bacteria, fungi [36], and protozoa [25,37]. In general, different mechanisms have been described to explain the activity of eugenol, including: (i) changes and cell morphology of cytoplasmatic membrane which increases membrane nonspecific permeability and affects the transport of ions and ATP; (ii) through increase of intracellular Reactive Oxygen Species (ROS) production which induces the inhibition of the growth of cell, disruption of the cell membrane and DNA damage resulting in cell decomposition and death; and (iii) inhibition of some enzymes such as protease, histidine carboxylase, amylase, and ATPase [36]. In particular, concerning *Leishmania* parasites, Hughes and collaborators have recently demonstrated that eugenol decreases lipid droplets without impacting membrane integrity and induces morphological alterations resulting in rounding and swelling promastigotes [25]. In this sense, further studies related to antileishmanial mechanisms of action should be addresses to confirm these predicted targets.

To promote eugenol as an antileishmanial potential lead, it should have acceptable physicochemical properties and pharmacokinetic profiles, which were analyzed through predicted drug-likeness properties and ADME characteristics [38]. In general, analysis of the ADME parameters showed good drug-like properties, which suggest that eugenol can be developed as an oral drug. Therefore, this compound was predicted to have good bioavailability and satisfied the drug likeliness parameters according to Lipinski (RO5), Ghose, Veber, Egan and Muegge rules. Thus, these theoretical data showed that eugenol presents drug-likeness properties the same or better than the currently used reference drug in antileishmanial therapy obtained from the Swiss ADME web tool, which provides free access to fast and robust models to compute the pharmacokinetics properties, drug-likeness and therapeutics chemistry of a molecule [38]. Recently, Daina and Zoete summarized the applications of this web tool in virtual screening to successfully support the discovery of bioactive small molecules [39]. In particular, the utility of the Swiss ADME tool has been shown in the components of EOs [40] and other natural compounds [41,42].

An interesting prediction of eugenol was related with penetration of eugenol through the Blood–Brain Barrier (BBB), which could suggest that eugenol can also exert its action in the Central Nervous System (CNS). In this sense, this compound could be promoted against other protozoal parasites, such as *Trypanosoma brucei*, *Plasmodium falciparum*, and *Toxoplasma gondii*, and may be suitable for treating the stages that compromise the CNS. In fact, the potential effect of eugenol against *Plasmodium falciparum* with an impact on cerebral malaria in an experimental murine model were recently reported [43]. In fact, accumulating studies have shown the beneficial effects of eugenol under various neuropathological conditions [44].

## 4. Materials and Methods

### 4.1. Plant Material

**Figure 3:**
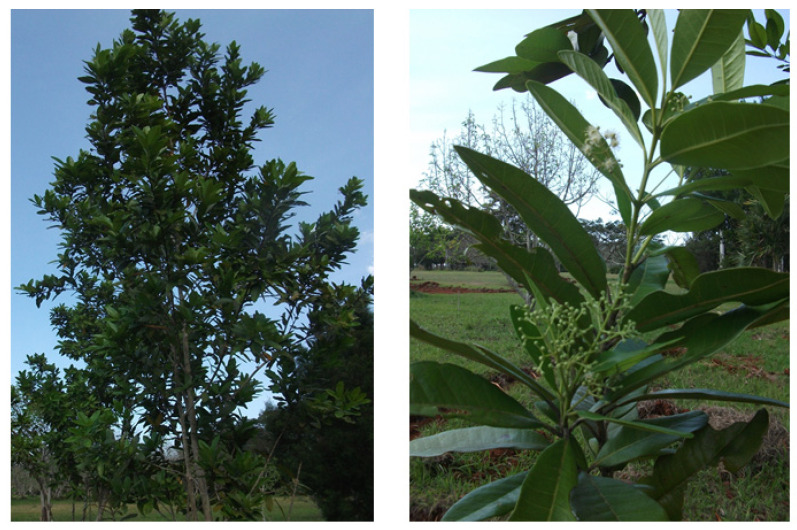
Photo of *Pimenta dioica* in the natural habitat at National Botanic Garden, Havana, Cuba (Photographs taken by the authors during the collection of the plant).

Aerial samples of *P. dioica* (Figure 3) were collected during the dry season (March) from the National Botany Garden of Havana (NBG; Havana, Cuba), located at 23°07′59.88″ N, 82°21′59.76″ W. A plant specimen was authenticated by MSc. Eldys Bécquer and deposited in the Herbarium of Cuban Flora (HFC), under the voucher number HFC-88584 of respective NBG. Vegetal material, collected early in the morning, was transported to Institute of Pharmacy and Food (Havana, Cuba) in plastic containers.

### 4.2. Essential Oil Extraction, Chemical Characterization, and Main Compound

In the same day, fresh leaves were selected, rinsed with abundant water, manually crushed into small pieces, and deposited in the flask of a Clevenger type apparatus. Immediately, the EO was obtained by hydrodistillation over a 5-h period. The essential oil was then chemically characterized by gas-chromatography coupled with a mass spectrometric detector (GC-MS) using a Shimadzu GC-MS-QP2010 Ultra Equipment (Shimadzu Scientific Instruments, Columbia, MD, USA), which was operated in the electron impact mode with electron energy = 70 eV, scan range = 40–400 atomic mass units, scan rate = 3.0 scans/s, and GC-MS solution software v. 4.45. The GC column was a ZB-5 fused silica capillary column (5% phenyl-polymethylsiloxane stationary phase, film thickness of 0.25 μm, a length of 30 m, and an internal diameter of 0.25 mm; Phenomenex, Torrance, CA, USA) and the carrier gas was helium with a column head pressure of 552 kPa, flow rate of 1.37 mL/min, injector temperature of 250 °C and the ion source temperature of 200 °C. The GC oven temperature program was programmed at 50 °C for initial temperature, and increased at a rate of 2 °C/min until 260 °C. The EO was prepared at 5% in a solution of CH_2_Cl_2_, which 0.1 μL was injected with a splitting mode (30:1). Finally, the essential oil components were identified according to their retention indices (RI) determined by reference to a homologous series of *n*-alkanes, and by comparison of their mass spectral fragmentation patterns with those reported in the literature [45], and data stored in our in-house Sat-Set library [46].

A sample of EO-Pd was used to perform the biological assays at 20 mg/mL. Additionally, eugenol (99% purity; Sigma-Aldrich, St. Louis, MO, USA) was dissolved at 40 mM. In both cases, dimethyl sulfoxide (DMSO; AppliedChem, Panreas, Germany) was used as the solvent.

### 4.3. Hierarchical Cluster Analysis

A total of 16 previously reported EO-Pd compositions [2,3,6,47,48,49,50,51,52,53,54,55] in addition to this present work were used for the Hierarchical Cluster Analysis (HCA). The 11 most abundant EO components (myrcene, *p*-cymene, limonene, 1,8-cineole, linalool, chavicol, eugenol, methyl eugenol, β-caryophyllene, α-humulene, and α-cadinol) were used to establish the chemical relationship between the EO samples using the XLSTAT software, version 2018.1.1.62926 (Addinsoft™, Paris, France). Euclidean distance was used to measure dissimilarity, and Ward’s method was used for cluster definition.

### 4.4. Parasite, Cells, Animals, and Reference Drug

*Leishmania amazonensis* (Strain code: MHOM/77BR/LTB0016) used in this study were isolated by aspiration with a needle from mouse lesions and cultivated at 26 °C in Schneider’s medium (Sigma-Aldrich) supplemented with 10% heat-inactivated fetal bovine serum (HFBS; Sigma-Aldrich) and antibiotics (100 μg of streptomycin/100 U of penicillin solution, Sigma-Aldrich). When promastigotes were obtained, passage was carried out every 3 or 5 days and used for experiments before to 10 in vitro passages.

Murine macrophage cells were also used, which were obtained from mouse peritoneum, washed with RPMI medium (Sigma, St. Louis, MO, USA) and antibiotics (100 μg of streptomycin/mL and 100 U of penicillin/mL; Sigma, St. Louis, MO, USA) at the moment of use from normal BALB/c mice.

Female BALB/c mice were used to obtain peritoneal macrophages and as animal model of CL. Mice were purchase from the National Center of Laboratory Animals Production (CENPALAB, Havana, Cuba) with their respective certificate of quality and were maintained under standard conditions with food and water ad libitum during the study. All of the experimental procedures involving animals were conducted in accordance with the Guide for the Care and Use of Laboratory Animals, Eighth Edition, and were approved by the Ethics Committee (CEI-IPK 44-20), Havana, Cuba.

As reference drug, Pentamidine^®^ (Richet, Buenos Aires, Argentina) was used. The compound was diluted in distilled water at 10 mg/mL.

### 4.5. In Vitro Antileishmanial and Cytotoxicity Assays

For antipromastigote viability test, a 96-well plate was used, which 100 µL of medium (Schneider’s medium + HFBS + antibiotics) was distributed in each well. In the last lane of the plate, additional 96 µL and 4 µL of stock solution of EO-Pd was added and six 1:2 serial dilutions were performed between lanes H to C. Then, 100 µL of exponentially growing parasites of *L. amazonensis* (10^6^ promastigotes/mL) in medium were added to each well (final concentration of DMSO was 1%), except lane A that was used as medium control; while lane B constituted the negative control (100% of growing). The plates were then sealed with Parafilm^®^ and incubated at 26 °C for 72 h. Afterward, 10 µL of resazurin solution (3 mM/PBS; Sigma-Aldrich) was added to each well and the plates were incubated for an additional 4 h. The absorbance was determined with a plate reader (Molecular Devices, San Jose, CA, USA) with a test wavelength of 560 nm and a reference wavelength of 600 nm from which median Inhibitory Concentrations (IC_50_) were calculated. The IC_50_ values were determined from linear dose–response curves fit to the data using a linear equation model. Each concentration of products was carried out in triplicate, and experiments were repeated three times. The results were expressed as means ± Standard Deviation (SD).

For the amastigote model, peritoneal macrophages were distributed in a 24-well plate with sterile slides at 10^6^ cells/mL in RPMI medium with 10% of HFBS and antibiotics. After 2 h of incubation at 37 °C and 5% CO_2_, the supernatant was eliminated and macrophages were infected with stationary-phase promastigotes of *L. amazonensis* at a 4:1 parasite/macrophage ratio for 4 h, at same conditions. Then, the free parasites were removed and four different concentrations of each product were added in a final volume of 1 mL (final concentration of DMSO was 1%). The plates were then incubated under the same conditions over 48 h [56]. Finally, the supernatant was discarded, cells were fixed with methanol, stained with Giemsa and microscopically examined. Total parasite burden was determined according to the number of infected macrophages and the number of amastigotes inside the macrophages of 25 cells [57]. IC_50_ values were determined from linear dose–response curves fit to the data using a linear equation model and results were expressed as means ± SD of three replicates.

Cytotoxicity was determined on uninfected peritoneal macrophages. Briefly, cells were collected and seeded at 3 × 10^5^ macrophages/mL in a 96-well plate (except lane A) and incubated at 37 °C and 5% CO_2_. After 2 h, the medium was removed and 50 µL of medium (RPMI + HFBS + antibiotics) was distributed in each well. In the last lane of the plate, additional 48 µL and 2 µL of products was added and six 1:2 serial dilutions were performed between lanes H to C. Then, 50 µL of medium were added to each well (final concentration of DMSO was 1%), except lane A that was used as medium control; while lane B constituted the negative control (100% of viability). The plate was incubated at the same conditions for 72 h and viability was determined with 15 µL of MTT solution (3-[4,5-dimethylthiazol-2-yl]-2,5-diphenyltetrazolium bromide; Sigma-Aldrich) at 5 mg/mL. After 4 h, formazan crystals were dissolved with 100 µL of DMSO and the optical density was measured at 570 nm and at 630 nm as reference wavelength [58]. In this case, median Cytotoxic Concentrations (CC_50_) were calculated from linear dose–response curves and results was expressed as means ± SD of three replicates, where lane A was used as medium control and lane B as 100% of macrophage viability.

Finally, Selectivity Indices (SI) were calculated through the ratio of CC_50_ (macrophages)/IC_50_ (amastigotes of *L. amazonensis*).

### 4.6. In Vivo Antileishmanial Assay

At day 0, female healthy BALB/c mice were infected in the right hind footpad with 5 × 10^6^ stationary-phase promastigotes of *L. amazonensis* by the subcutaneous route in a volume of 50 μL. The animals were maintained in standard conditions until 4 weeks post-infection (p.i.), which mice were randomly distributed into four groups (*n* = 8 in each group): Group 1, EO-Pd (30 mg/kg); Group 2, eugenol (30 mg/kg); Group 3, Pentamidine^®^ (4 mg/kg); Group 4, placebo (solvent used to dilute studied products: 30% DMSO:70% saline solution [59]); Group 5, control (untreated). Treatments with EO-Pd and eugenol were applied every four days to a total of five doses by the intralesional route, while Pentamidine^®^ was administered by intraperitoneal route for 15 days. During the experimental period, animals were observed daily, body weight and the lesion size (mean of the differences between infected and uninfected footpads) were determined weekly using a technical bascule (SCALTEC, Göttingen, Germany) and a caliper was used to measure footpad swelling and lesion diameter. In addition, parasite burden was determined at the end of treatment (6 weeks p.i.) and in the final point (10 weeks p.i.) through the culture microtitration method in 96-well plates using excised tissues of subcutaneous infected area [60].

### 4.7. In Silico Predictions

The chemical structure of eugenol was constructed using BIOVIA Draw Program 2018 (Version 18.1.0.1601, Dassault Systémes). After that, SMILES of structure were inserted in the online server for a Prediction of Activity Spectra for Substances (PASS; accessed on 28 July 2022) and automatically potential bioactivity predictions were obtained. Activities with *Pa* > 0.7 (probability of the substance to be active) were selected. In parallel, SMILES was also used to obtain its physicochemical properties and Absorption, Distribution, Metabolism, Excretion (ADME) from SwissADME online server (accessed on 28 July 2022) [38] and compare with pentamidine as reference drug. In all cases, data were exported to Excel Microsoft 2010, store in a spreadsheet and analyzed.

### 4.8. Statistical Analysis

In the in vitro assays, respective IC_50_ and CC_50_ were compared using Mann–Whitney. In the in vivo assay, a Variance Analysis Test, accompanied by a Post Hoc Test (Fisher’s least significant difference (LDS test) or planned comparison) was applied for comparison among groups [61]. In both cases, Statistica Program for Windows (Version 13.1, StatSoft, Inc. 2016) was used and a *p* < 0.05 were considered statistical differences.

## 5. Conclusions

This study showed for first time the potentialities of the EO-Pd to inhibit *L. amazonensis* (promastigotes and intracellular amastigotes) growth with a selective activity and demonstrated the antileishmanial effect in a murine model of experimental CL. This interesting result could be linked to the activity of the major component eugenol, which displayed a potent in vivo efficacy and good drug-like properties that could contribute to the discovery of new effective drugs against leishmaniasis.

## Figures and Tables

**Table 1:** Peak assignment for gas chromatography—mass spectrometry profiles of the essential oil obtained by hydrodistillation from *Pimenta dioica* leaves collected from the National Botanic Garden, Havana, Cuba.

RI ^1^	Compound	%
873	(3*Z*)-Hexenol	0.1
880	1-Hexanol	tr ^2^
924	α-Thujene	0.3
930	α-Pinene	0.3
971	Sabinene	tr
987	Myrcene	0.2
1000	α-Phellandrene	0.6
1006	δ-3-Carene	0.1
1012	1,4-Cineole	tr
1013	α-Terpinene	0.2
1021	*p*-Cymene	3.6
1026	β-Phellandrene	0.6
1028	1,8-Cineole	2.3
1059	γ-Terpinene	0.1
1090	Terpinolene	0.6
1104	Linalool	0.1
1150	Unidentified	0.1
1180	Terpinen-4-ol	0.8
1189	*p*-Cymen-8-ol	0.2
1193	α-Terpineol	0.2
1201	Methyl chavicol (=Estragole)	tr
1203	*cis*-Sabinol	0.1
1253	Chavicol	tr
1286	Thymol	tr
1290	Carvacrol	tr
**1360**	**Eugenol**	**85.1**
1390	β-Elemene	0.1
1395	Vanillin	0.1
1416	β-Caryophyllene	0.7
1450	α-Humulene	0.4
1484	β-Selinene	0.2
1494	α-Selinene	0.3
1525	δ-Cadinene	0.1
1581	Caryophyllene oxide	0.8
1590	Viridiflorol	0.1
1602	Ledol	0.1
1608	Humulene epoxide II	0.3
1613	Unidentified	0.2
1616	Junenol	0.1
1628	1-*epi*-Cubenol	0.1
1641	τ-Cadinol	0.2
1643	τ-Muurolol	0.1
1647	α-Muurolol	0.1
1654	α-Cadinol	0.3
1658	Selin-11-en-4β-ol	0.6
	**Total identified**	**99.7**

^1^ Retention Index determined with respect to a homologous series of *n*-alkanes on a ZB-5 column. ^2^ Traces (concentration < 0.05%). Bold letters indicate main compound.

**Table 2:** In vitro antileishmanial activity, cytotoxic effects, and selectivity of the leaf essential oil of *Pimenta dioica* (collected from the National Botanic Garden, Havana, Cuba) and reference drug.

Products	IC_50_^1^ ± SD ^2^ (µg/mL)		CC_50_^3^ ± SD (µg/mL)	SI ^4^
	Promastigotes	Amastigotes		
*P. dioica* EO	9.7 ± 0.7	11.3 ± 2.1	104.5 ± 0.9	9
Pentamidine ^5^	2.6 ± 0.9	1.3 ± 0.1	11.7 ± 1.7	9

^1^ Median inhibitory concentration. ^2^ Standard deviation. ^3^ Median cytotoxic concentration. ^4^ Selectivity index (CC_50_ macrophage / IC_50_ amastigotes of *L. amazonensis*). ^5^ Reference drug.

**Table 3:** Prediction of biological activities and ligand interactions by the PASS online webserver of eugenol, the main compound identified in the essential oil from *Pimenta dioica* collected in National Botanic Garden, Havana, Cuba (organized by *Pa* value).

Prediction		Eugenol	
		*Pa* ^1^	*Pi* ^2^
**Biological Activity:**	Carminative	0.941	0.001
	Antimutagenic	0.878	0.003
	Antieczematic	0.868	0.008
	Mucomembranous protector	0.835	0.011
	Preneoplastic conditions treatment	0.803	0.004
	Anesthetic general	0.742	0.005
	Antiseptic	0.722	0.005
**Ligand interactions:**	Aspulvinone dimethylallyltransferase inhibitor	0.937	0.004
	Chlordecone reductase inhibitor	0.902	0.005
	Feruloyl esterase inhibitor	0.881	0.005
	Caspase 3 stimulant	0.873	0.004
	JAK2 expression inhibitor	0.873	0.004
	Linoleate diol synthase inhibitor	0.863	0.004
	CYP2E1 substrate	0.856	0.004
	Membrane integrity agonist	0.866	0.020
	CYP2E substrate	0.850	0.004
	Vanillyl-alcohol oxidase inhibitor	0.840	0.002
	CYP2A substrate	0.841	0.005
	Ubiquinol-cytochrome-c reductase inhibitor	0.825	0.024
	G-protein-coupled receptor kinase inhibitor	0.807	0.012
	Beta-adrenergic receptor kinase inhibitor	0.807	0.012
	MMP9 expression inhibitor	0.797	0.003
	Gluconate 2-dehydrogenase (acceptor) inhibitor	0.797	0.017
	Membrane permeability inhibitor	0.781	0.013
	5 Hydroxytryptamine release stimulant	0.773	0.017
	Apoptosis agonist	0.743	0.011
	Cardiovascular analeptic	0.736	0.005
	MAP kinase stimulant	0.735	0.004
	CYP2C8 inhibitor	0.733	0.004
	Fatty-acyl-CoA synthase inhibitor	0.724	0.010
	CYP2C substrate	0.725	0.015
	TP53 expression enhancer	0.724	0.021
	Respiratory analeptic	0.715	0.014
	CYP1A2 substrate	0.709	0.008
	HMOX1 expression enhancer	0.705	0.008
	CYP1A substrate	0.701	0.011
	CYP2C12 substrate	0.734	0.051
	CDP-glycerol glycerophosphotransferase inhibitor	0.717	0.049

^1^ Probability to be active (*Pa* > 0.7). ^2^ Probability to be inactive.

**Table 4:** Drug-likeness and ADME properties predicted by in silico studies using SwissADME online webserver of eugenol, the main compound identified in the leaf essential oil from *Pimenta dioica* collected from the National Botanic Garden, Havana, Cuba.

Predicted Parameter	Eugenol	Pentamidine
**Physico-Chemical Properties:**		
Molecular weight	164.2	340.4
Hydrogen bond acceptors	2	4
Hydrogen bond donors	1	4
Number of rotatable bonds	3	10
Topological polar surface area (Å)	29.5	118.2
Molar refractivity	49.1	100.7
**Absorption Parameters:**		
Consensus LogS	−2.46	−3.26
Consensus Log P	2.25	2.72
Solubility class	Soluble	Soluble
**Drug Likeness Prediction:**		
Lipinski (RO5)	0	0
Ghose	0	0
Veber	0	0
Egan	0	0
Muegge	1	0
**Bioavailability**		
Bioactivity score	0.55	0.55
Synthetic accessibility	1.58	2.38
**Distribution Parameters Prediction**		
Log Kp (cm/seg)	−5.69	−6.56
GI Absorption	High	High
BBB Permeant	Yes	No
**Metabolism Parameters Prediction**		
P-Glycoprotein substrate	No	No
CYP1A2, CYP2C19, CYP2C9, CYP2D6 and CYP3A4 inhibitors	No, except to CYP1A2	No, except to CYP2C9 and CYP2D6

## Data Availability

All data are available in the article and Supplementary Materials.

## Supplementary Materials

The following supporting information can be downloaded at: https://www.mdpi.com/article/10.3390/ph17010064/s1, Figure S1: Gas chromatogram of *Pimenta dioica* essential oil.
