# Supplementary figures and images for: Eugenol-Rich Essential Oil from Pimenta dioica: In Vitro and In Vivo Potentialities against Leishmania amazonensis
Source: Pharmaceuticals (Basel). 2023 Dec 29;17(1):64. doi: 10.3390/ph17010064 (PMC10819736; doi:10.3390/ph17010064)

**Supplementary Figure S1.** Gas chromatogram of *Pimenta dioica* essential oil.

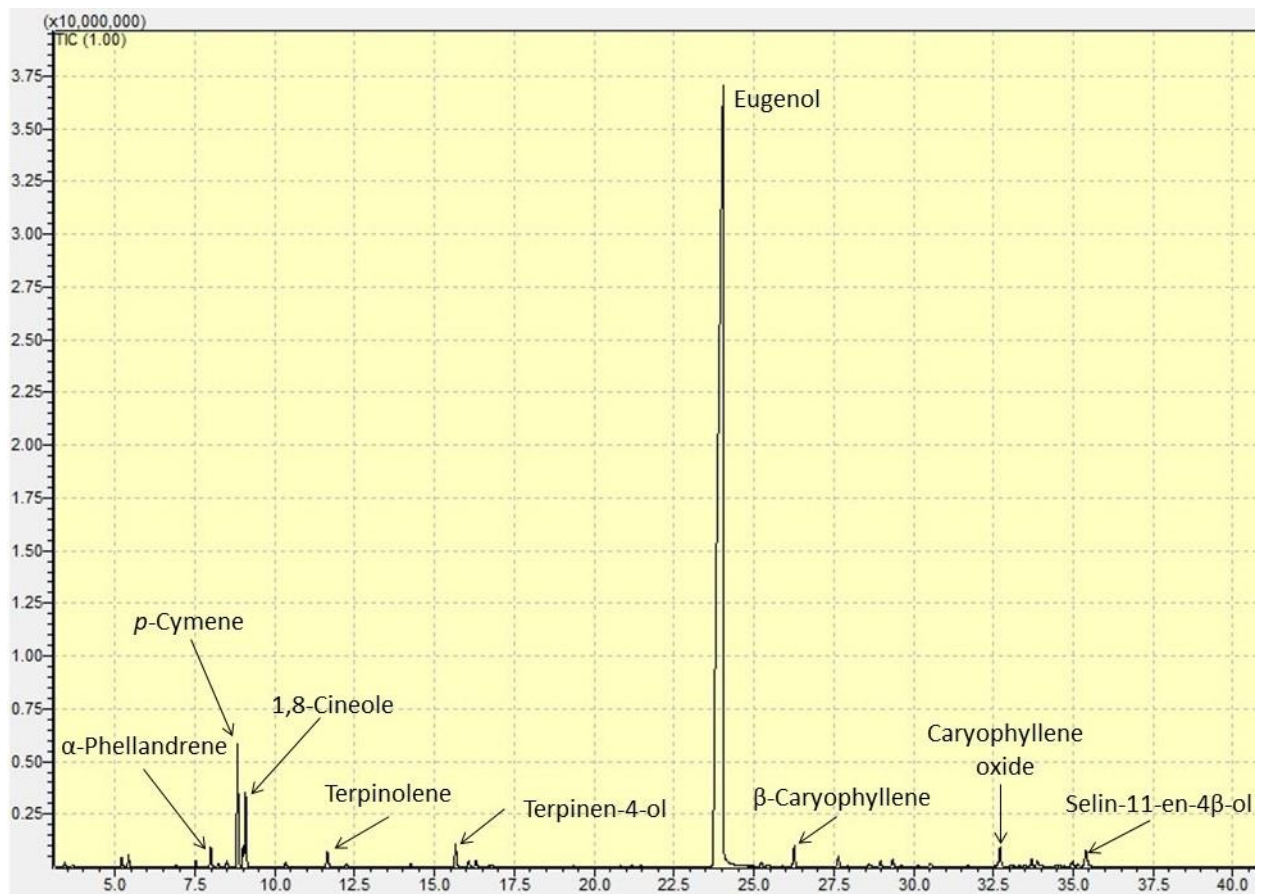

Supplement: Supplementary file 1 [file pharmaceuticals-17-00064-s001.zip › pharmaceuticals-2723657-supplementary.pdf]
